# TFPIα anticoagulant function is highly dependent on protein S in vivo

**DOI:** 10.1126/sciadv.adk5836

**Published:** 2024-02-02

**Authors:** Anastasis Petri, Parvathy Sasikumar, Patricia Badia Folgado, David Jones, Yaoxian Xu, Josefin Ahnström, Isabelle I. Salles-Crawley, James T. B. Crawley

**Affiliations:** ^1^ Centre for Haematology, Hammersmith Hospital Campus, Imperial College London, London, UK.; ^2^ Vascular Biology Research Centre, Molecular and Clinical Sciences Research Institute, St. George's University of London, London, UK.

## Abstract

Tissue factor pathway inhibitor α (TFPIα) is the major physiological regulator of the initiation of blood coagulation. In vitro, TFPIα anticoagulant function is enhanced by its cofactor, protein S. To define the role of protein S enhancement in TFPIα anticoagulant function in vivo, we blocked endogenous TFPI in mice using a monoclonal antibody (14D1). This caused a profound increase in fibrin deposition using the laser injury thrombosis model. To explore the role of plasma TFPIα in regulating thrombus formation, increasing concentrations of human TFPIα were coinjected with 14D1, which dose-dependently reduced fibrin deposition. Inhibition of protein S cofactor function using recombinant C4b-binding protein β chain significantly reduced the anticoagulant function of human TFPIα in controlling fibrin deposition. We report an in vivo model that is sensitive to the anticoagulant properties of the TFPIα-protein S pathway and show the importance of protein S as a cofactor in the anticoagulant function of TFPIα in vivo.

## Teaser

The anticoagulant function of TFPIα is highly dependent on protein S cofactor function in vivo.

## INTRODUCTION

The initiation of coagulation is regulated by tissue factor pathway inhibitor (TFPI) (*1*). In humans, TFPI exists in two major alternatively spliced isoforms, TFPIα and TFPIβ (*2*). TFPIα consists of three Kunitz (K) domains (K1 to K3) followed by a highly basic C-terminal tail. Full-length TFPIα circulates at low concentrations (0.2 to 0.5 nM) but can also be acutely/locally released by activated platelets (*3*). Smaller truncated forms of TFPI also circulate, primarily in association with plasma lipoproteins, but with reduced inhibitory function (*4*). As a soluble protein, full-length TFPIα exerts its function both in plasma and on cell surfaces exposed to the plasma. The inhibitory function of TFPIβ, which contains only the K1 and K2 domains that are glycosylphosphatidylinositol-anchored to the cell surface, is restricted to the surface of cells expressing this isoform (*5*). In both TFPIα and TFPIβ, K1 and K2 domains directly inhibit the tissue factor (TF)–activated factor VII (FVIIa) complex and activated factor X (FXa), respectively. In TFPIα, the K3 domain interacts with its cofactor, protein S, which enhances the rate of FXa inhibition 4- to 10-fold and, therefore, augments its anticoagulant function (*6**–**10*). This cofactor function is thought, at least in part, to be attributed to the ability of protein S to augment the association of TFPIα to the phospholipid surfaces upon which the initiation of coagulation occurs (*6**–**11*). More recently, the alternatively spliced form of FV, FV-short, has been revealed to bind the TFPIα C-terminal tail with high affinity via an acidic region in the truncated B domain (*12*). The C terminus of TFPIα is also needed for the efficient inhibition of FXa (*13*) and also binds to FVa that retains portions of its B domain, allowing it to inhibit early forms of prothrombinase (*14*). The interaction of TFPIα with FV-short not only reduces the rate of renal filtration of TFPIα and so controls the levels of circulating TFPIα (*12*) but also functions as a synergistic cofactor in enhancing TFPIα anticoagulant function in a protein S–dependent manner (*15**–**17*).

Although TFPIα function has been comparatively well characterized in vitro, the role/importance of the TFPIα anticoagulant pathway and its dependency on protein S in vivo is less well understood. The reasons why the physiological importance of protein S–dependent TFPIα enhancement is not well characterized is, in part, due to the embryonic lethality of both *Tfpi^−/−^* and *Pros1*^−/−^ mice, which hampers the analysis of TFPI and protein S function in adult mice (*18**–**20*). Severe TFPI deficiency in mice causes consumptive coagulopathy associated with unregulated TF function (*18*). *Tfpi^−/−^* embryonic lethality can be rescued by crossing mice onto a low human TF background, consistent with the primary/major role of TFPI in regulating TF-dependent functions (*21*). Alternatively, maintenance of trophoblast expression of TFPI in TFPI-null embryos can also rescue the developmental/placental defect but result in mice with a prothrombotic tendency (*22*). Because of the role of protein S as a cofactor for both TFPIα and for activated protein C, *Pros1^−/−^* mice lethality may represent the consequence of impairment of two distinct anticoagulant pathways (*19*, *20*). Protein S deficiency in mice is slightly more severe (embryonic) than protein C deficiency (neonatal), which may either reflect differences in transfer of maternal protein C and protein S across the placenta, and/or additional roles of protein S beyond the protein C pathway (*19*, *20*, *23*).

There are also some important interspecies differences in both TFPI and protein S between humans and mice. These include the markedly reduced levels (~60 pM) of full-length TFPIα in murine plasma (primarily present in murine platelets) and the presence of an additional plasma isoform not found in humans, TFPIγ, which contains only K1 and K2 and which circulates at much higher concentrations (~30 nM) (*24**–**26*). Studies on TFPI isoform–specific deletion in mice have revealed that TFPIα deficiency in mice does not influence hemostatic plug formation in the laser injury model (*27*). Given that inhibition of all TFPI isoforms increases fibrin deposition in this model (*28*), this suggests that TFPIβ and TFPIγ are responsible for much of the TFPI-dependent hemostatic control in mice. This may also suggest that the protein S–dependent TFPIα enhancement may be of lesser importance in mice than in humans.

A further difference between humans and mice is that, in humans, ~60% of plasma protein S circulates in tight complex with C4b-binding protein (C4BP). Only free protein S exerts TFPIα cofactor function, likely due to overlapping binding sites between C4BP and TFPI K3 in protein S (*6*, *15*, *29*). C4BP is an octameric protein consisting of either eight covalently linked C4BP-α chains or seven C4BP-α chains and one C4BP-β chain (*30*). Only β chain–containing C4BP binds protein S. Mice do not express C4BP-β chain (*31*). Consequently, all protein S (~150 nM) in mice is free.

Together, the differences between humans and mice make it challenging to probe protein S–TFPIα anticoagulant pathway in a murine system. However, given that murine models of thrombosis are well established, we aimed to develop and characterize tools and approaches to formally assess the contribution of protein S cofactor function to the anticoagulant function of TFPIα in vivo.

## RESULTS

### Ex vivo analysis of TFPI-protein S anticoagulant function

**Fig. 1.**
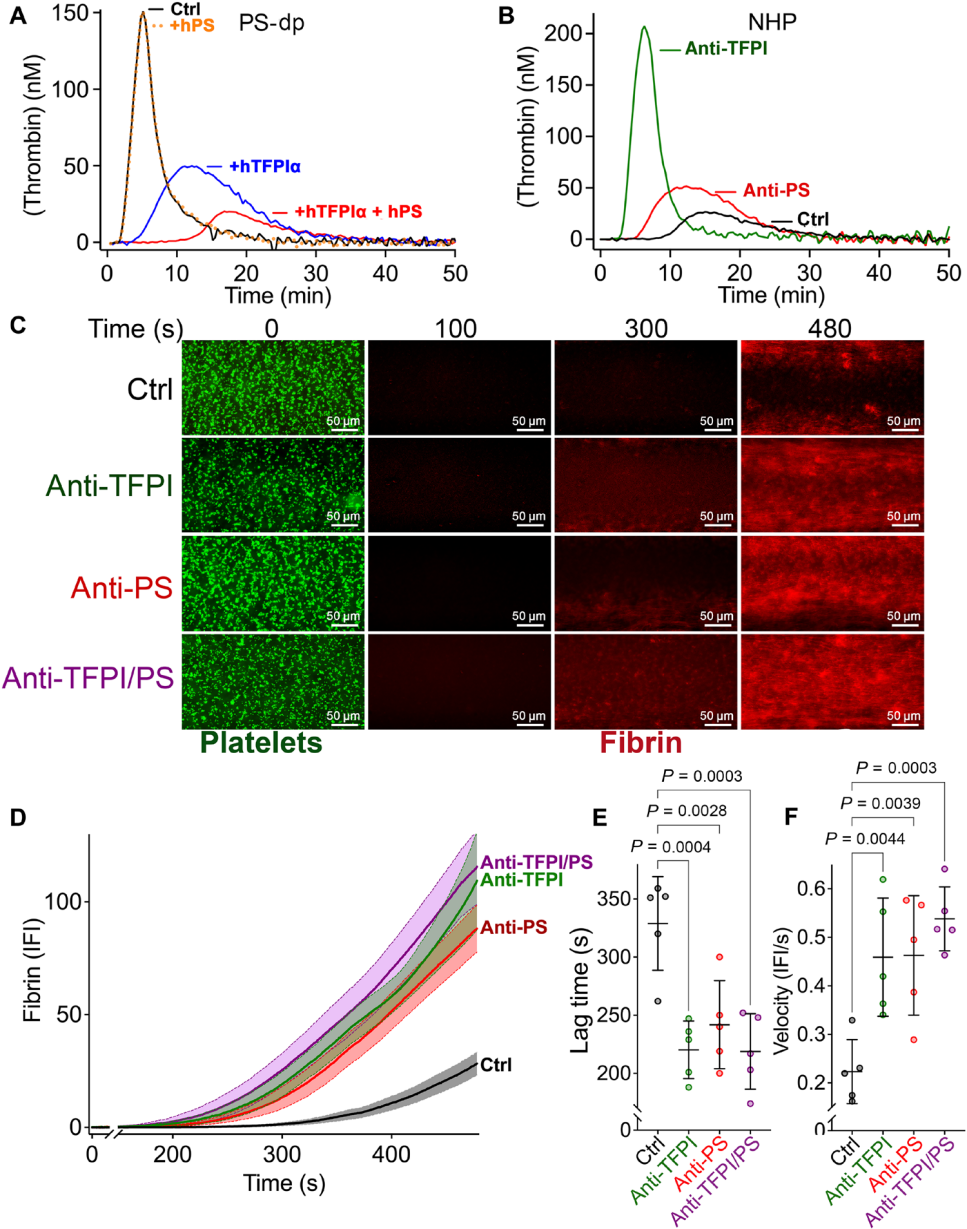
TFPIα cofactor function of protein S in CAT and microfluidic assays. (**A**) Thrombin generation was initiated using 1 pM TF in protein S–depleted plasma (PS-dp) (which is codepleted of TFPIα) and analyzed by CAT (Ctrl, black). Wells also containing either 50 nM human protein S (+PS, orange dashed), 1 nM human TFPIα (+TFPIα, blue), or both (+TFPIα + PS, red) were analyzed in parallel (graph representative of *n* = 3). (**B**) Thrombin generation was initiated using 1 pM TF in normal human plasma (NHP) and analyzed by CAT (Ctrl, black). Wells also containing either inhibitory anti-protein S (anti-PS, red) or inhibitory anti-TFPI (green) antibodies were analyzed in parallel (graph representative of *n* = 3). (**C**) Microfluidic channels were coated with 50 nM human VWF and 1 pM TF. Thereafter, citrated whole blood containing DiOC_6_ to label platelets (green) was perfused to capture a single layer of platelets. Channels were washed and perfused with recalcified plasma containing Alexa 594–labeled fibrinogen (red) with and without the addition of anti-TFPI, anti-protein S, or anti-TFPI and anti-protein S antibodies. Fibrin deposition was recorded in real time by fluorescence microscopy. Representative micrographs at different time points (0 to 480 s) are presented. (**D**) Graphical representation of the mean (± SEM) integrated fibrin fluorescent intensity (IFI) over time (*n* = 5) in the absence (Ctrl, black) and presence of anti-TFPI (green), anti-protein S (anti-PS, red), or anti-TFPI and anti-protein S (anti-TFPI/PS, purple) antibodies. (**E**) Graphical representation of the mean lag time (± SEM) for data presented in (D). (**F**) Graphical representation of the rate of fibrin deposition (±SEM) for data presented in (D). Data in (E) and (F) were compared by one-way analysis of variance (ANOVA) with Dunnett’s multiple comparison test; *P* values <0.05 were considered significant.

The ability of protein S to augment the anticoagulant function of TFPIα in vitro is well described (*7*, *8*, *10*, *11*, *15*, *17*, *29*, *32*). Using calibrated automated thrombography (CAT) assays in protein S–depleted plasma, which also lacks TFPIα (*15*), 1 pM TF induces appreciable and rapid thrombin generation (Fig. 1A). Whereas addition of 50 nM protein S has no effect on this, 1 nM TFPIα significantly extends the lag time and reduces the peak height, which is further enhanced by the addition of both 1 nM TFPIα and 50 nM protein S. Similarly, in normal human plasma, the lag time is shortened by the addition of anti–protein S antibodies that inhibit its TFPIα cofactor function (Fig. 1B). When TFPI anticoagulant function is completely blocked using anti-TFPI antibodies, 1pM TF induces thrombin generation with appreciably higher peak height and a short lag time. Although these assays reveal the anticoagulant actions of TFPIα in plasma and the ability of protein S to augment this function, they are potentially difficult to extrapolate to the physiological setting. TFPIα is normally present at low concentrations (0.2 to 0.5 nM) and so has the potential to be significantly depleted by FXa, which may reduce the extent of its inhibitory function as measured in CAT assays. Moreover, in CAT assays, coagulation occurs in suspension on phospholipid vesicles that are optimized for coagulation factor function rather than on activated cell/platelet surfaces under flow at sites of vessel damage. Although CAT assays can be modified to use platelets rather than phospholipid vesicles, which can therefore include the contributions of platelet TFPIα and protein S (*11*), these remain in suspension rather than adhering to and aggregating on a surface. Together, these differences may potentially under/overestimate the anticoagulant function of TFPIα in CAT assays. We therefore optimized a microfluidic assay to monitor coagulation under flow over a two-dimensional surface. Unlike previous microfluidic assays, we used a two-stage approach (*33*). Microfluidic channels were coated with both 50 nM von Willebrand factor (VWF; rather than collagen) and 20 pM TF. We first captured a monolayer of platelets by perfusing anticoagulated whole blood through the channels at high shear (Fig. 1C). Capturing platelets on VWF resulted in a platelet monolayer that was not activated before initiation of coagulation. Thereafter, channels were washed before perfusion of recalcified plasma at low shear containing fluorescent fibrinogen. Coagulation was initiated by the TF on the microchannel surface and propagated on the surface of the platelet layer activated by locally generated thrombin. Fibrin deposition was monitored in real time by video fluorescence microscopy (Fig. 1, C and D). Addition of inhibitory anti–protein S, anti-TFPI, or both resulted in a marked increase in fibrin deposition (Fig. 1, C and D), characterized by a significant shortening of the lag time (Fig. 1E) as well as a marked enhancement of the rate of fibrin deposition (Fig. 1F). In this assay, blocking protein S had a very similar effect to blocking TFPI alone or both TFPI and protein S, suggesting that under flow on the platelet surface, the TFPIα function is highly dependent on protein S.

### Generation and characterization of inhibitory anti-murine TFPI monoclonal antibody

**Fig. 2.**
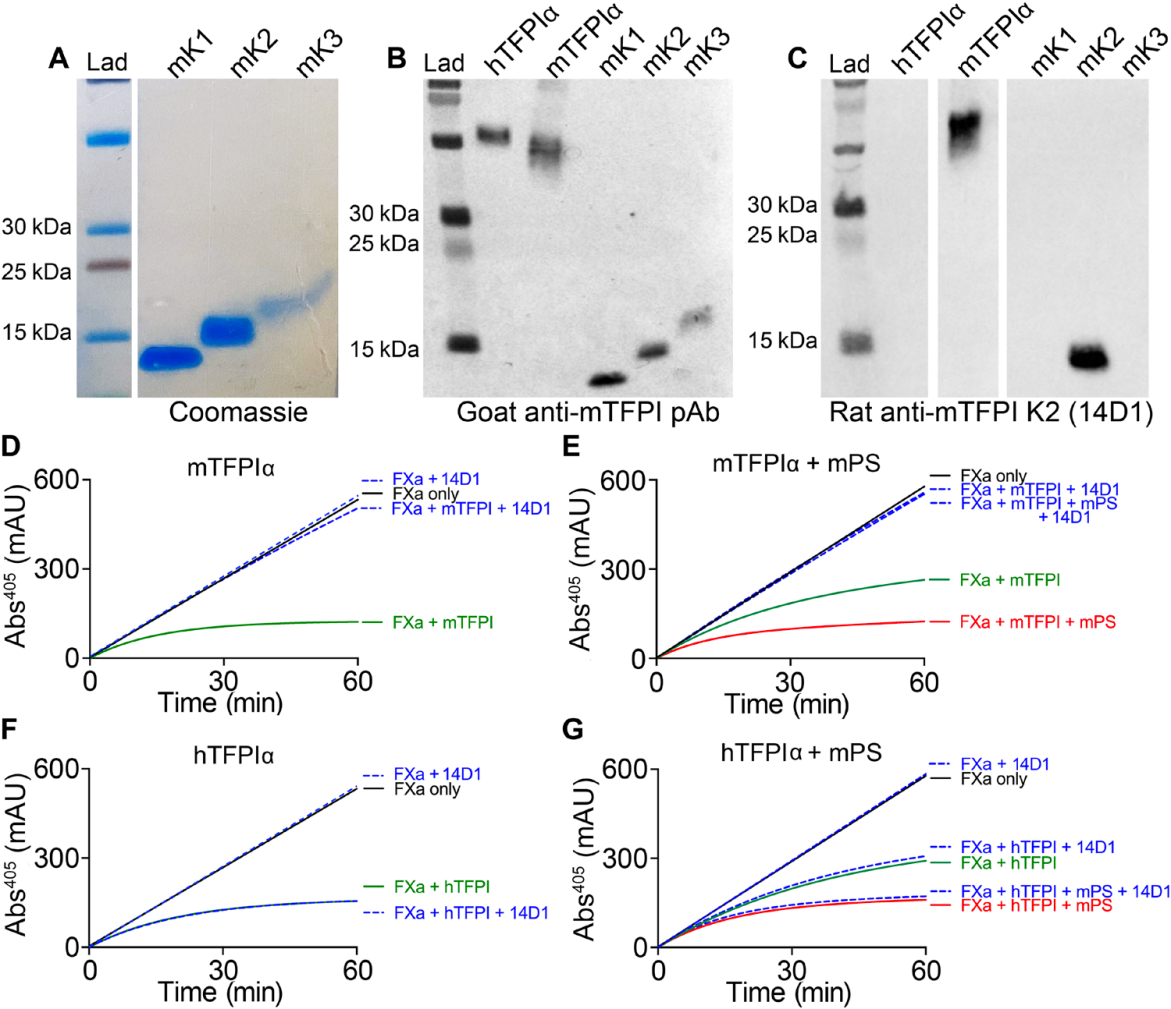
Generation and characterization of inhibitory rat anti-murine TFPI antibody (14D1). (**A**) Recombinant murine (m)TFPIα K domains mK1, mK2, and mK3 were expressed in insect cells and purified after analysis by SDS–polyacrylamide gel electrophoresis (SDS-PAGE PAGE) and Coomassie staining. (**B**) Western blot analysis (nonreducing) of a goat anti-mTFPI polyclonal antibody recognizes mTFPIα and mK1, mK2, and mK3. This also cross-reacts with human (h)TFPIα. (**C**) By Western blot analysis (nonreducing), the rat anti-mTFPI monoclonal antibody (14D1) specifically recognizes mTFPIα and mK2 (but not mK1 or mK3) and does not cross-react with hTFPIα. (**D** to **G**) FXa inhibition assays reporting chromogenic substrate cleavage measured by changes in absorbance at 405 nm (mAU) over time. (D) Representative FXa activity assays (*n* = 3) containing 0.5 nM FXa performed in the absence (black) and presence of 8 nM mTFPIα (green) both ± 80 nM 14D1 (blue dashed). (E) Representative FXa activity assays (*n* = 3) containing 0.5 nM FXa performed in the presence of 2 nM mTFPIα (green) or 2 nM mTFPIα and 50 nM murine protein S (mPS; red) both ± 80 nM 14D1 (blue dashed). (F) FXa inhibition assay performed as in (D) except using 8 nM hTFPIα. (G) FXa inhibition assay performed as in (E) except using 2 nM hTFPIα.

To specifically analyze the anticoagulant function of TFPIα in vivo, we aimed to inhibit all endogenous isoforms of murine TFPI (TFPIα, TFPIβ, and TFPIγ) and then examine the anticoagulant function of human TFPIα injected into mice. We purified the recombinant murine K1, K2, and K3 domains (Fig. 2A) to test the specificity of a commercially available polyclonal anti-murine TFPI antibody. Although this recognized murine TFPIα and each of the three isolated K domains, it also cross-reacted with human TFPIα (Fig. 2B). We instead developed a rat monoclonal antibody (14D1) that recognized murine TFPIα and murine K2 domain but did not cross-react with human TFPIα (Fig. 2C). We demonstrated that 14D1 completely blocked the ability of murine TFPIα to inhibit FXa in vitro (Fig. 2D) both in the absence and in the presence of murine protein S (Fig. 2E). When the same FXa activity assays were performed containing human TFPIα, 14D1 had no effect (Fig. 2, F and G), confirming the specificity of 14D1 for functional inhibition of murine (and not human) TFPI.

**Fig. 3.**
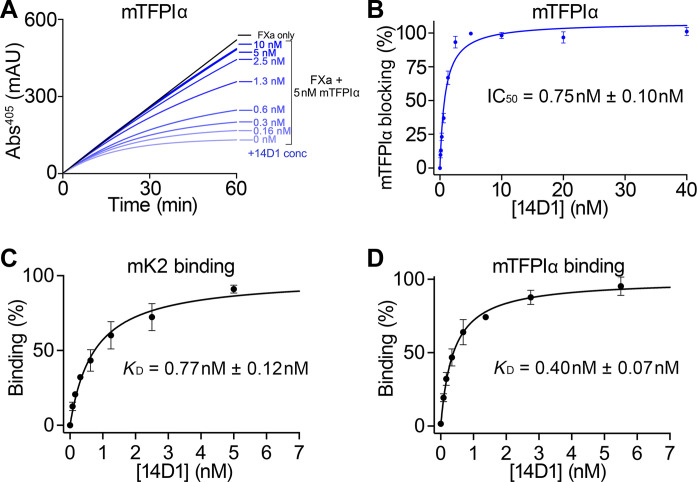
Quantitation of 14D1 TFPI inhibitory/binding function. (**A**) FXa inhibition assays reporting chromogenic substrate cleavage measured by changes in absorbance at 405 nm (mAU) over time. Representative FXa inhibition assay (*n* = 3) containing 0.5 nM FXa (black) ± 5 nM mTFPIα with increasing concentrations of 14D1 (0 to 80 nM, but only 0 to 10 nM plotted; blue). (**B**) Data from (A) were plotted as mTFPIα blocking (the FXa activity at 30 mins with 0 nM 14D1 was defined as 0% blocking, and 100% was the activity of FXa without mTFPIα) as a function of 14D1 concentration (*n* = 3). From this, the mean IC_50_ ± SD for blockade of mTFPIα inhibitory function was derived. (**C** and **D**) Affinity of 14D1 for recombinant mK2 (C) or mTFPIα (D) was measured by adsorption of mK2 or mTFPIα, respectively, onto microtiter wells and titration of increasing concentrations of 14D1 followed by detection using HRP-labeled anti-rat IgG. Data presented are mean-normalized binding ± SD (*n* = 3). From these, the mean binding affinity (*K*_D_) ± SD of 14D1 was derived.

To more specifically evaluate the potency of 14D1 to block murine TFPIα function, we performed FXa activity assays in the presence of 5 nM murine TFPIα into which we titrated increasing concentrations of 14D1 (Fig. 3A—Note that because of the bivalent nature of antibodies, >90% inhibition of 5 nM TFPIα was achieved by 2.5 nM 14D1). From these assays, we derived a median inhibitory concentration (IC_50_) of 0.75 ± 0.1 nM, indicative of high-affinity inhibition (Fig. 3B). To confirm this, we performed binding assays using both the isolated murine K2 domain (Fig. 3C) and murine TFPIα (Fig. 3D), from which we derived a dissociation constant (*K*_D_) of 0.77 ± 0.12 nM and 0.40 ± 0.07 nM, respectively, consistent with a high-affinity interaction and effective functional inhibition of TFPIα.

### Anticoagulant function of human TFPIα in laser-induced thrombosis

**Fig. 4.**
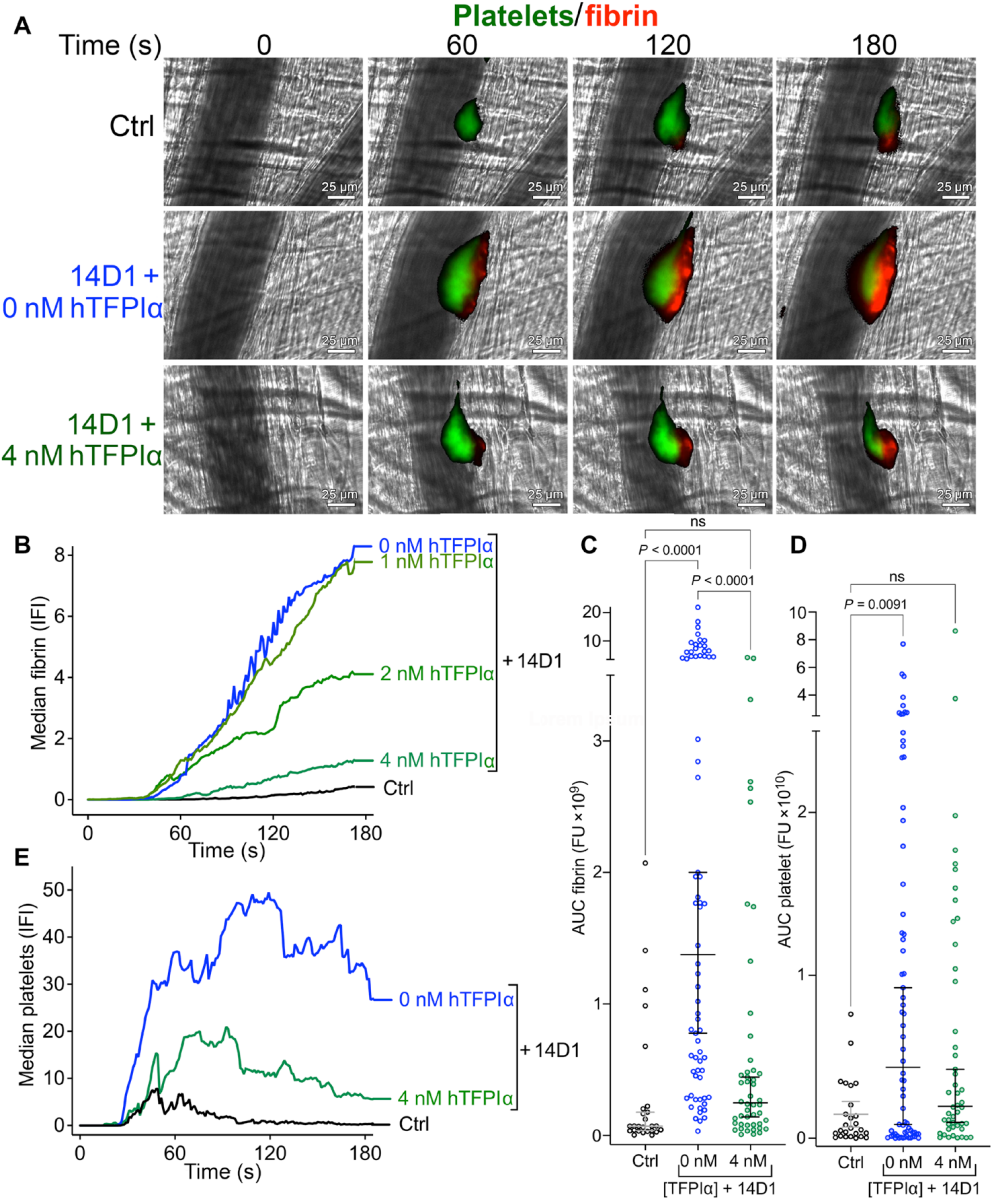
Inhibitory function of hTFPIα in vivo. Laser-induced thrombosis in murine cremaster muscle arterioles was performed using a mild laser injury following injection of anti–GPIbβ–DyLight 488 antibody to label platelets (green) and Alexa 647–labeled human fibrinogen (red) to label fibrin. Thrombus formation was monitored in real time by intravital microscopy. (**A**) Representative images of the mean fibrin deposition at 0 to 180 s in mice (C57Bl6/J) injected with control rat IgG (Ctrl), inhibitory rat anti-mTFPIα (14D1) ± 4 nM recombinant hTFPIα—Images are representative of the median data presented in (B) (see also accompanying movie S1). (**B**) Median fibrin deposition (IFI) over time in mice injected with control rat IgG (Ctrl, black; *n* = 26), inhibitory rat anti-mTFPI (14D1, blue; *n* = 70) ± 1 nM hTFPIα (*n* = 12), 2 nM hTFPIα (*n* = 12), or 4 nM hTFPIα (green; *n* = 48). (**C**) For all thrombi, the total fibrin deposition (represented by the area under the curve, AUC fibrin) is plotted. Individual data are plotted with median ± 95% confidence interval. Data were compared by ANOVA with a Dunn’s multiple comparison test; *P* values <0.05 were considered significant; ns, not significant. (**D**) For all thrombi, the total platelet deposition (represented by the area under the curve, AUC platelet) is plotted. Individual data are plotted with median ± 95% confidence interval. Data were compared by ANOVA with a Dunn’s multiple comparison test; *P* values <0.05 were considered significant. FU, fluorescence units. (**E**) Median platelet deposition (IFI) over time in mice injected with control rat IgG (Ctrl, black; *n* = 26), inhibitory rat anti-mTFPI (14D1, blue; *n* = 70) ± 4 nM hTFPIα (green; *n* = 48).

To analyze the TFPI anticoagulant pathway in vivo, we used a laser injury model of cremaster muscle arterioles. Using this injury model that initiates development of small nonocclusive thrombi with little/no collagen exposure, mice injected with control rat immunoglobulin G (IgG) developed small thrombi with modest fibrin deposition (Fig. 4, A to E). Conversely, mice injected with 14D1 that blocks the K2 domain present in all endogenous murine TFPI isoforms (TFPIα, TFPIβ, and TFPIγ) resulted in a significant increase in both the amount of fibrin deposition (Fig. 4, B and C) and thrombus size (Fig. 4, D and E, measured by platelet accumulation). We then titrated in increasing concentrations of human TFPIα (1 to 4 nM) in combination with 14D1, which resulted in a dose-dependent reduction in fibrin accumulation (Fig. 4B). At 4 nM human TFPIα, fibrin and platelet accumulation were similar to control mice (Fig. 4, A to E), demonstrating the sensitivity of the laser injury model to the anticoagulant function of human TFPIα (also see movie S1). The requirement for supraphysiological concentrations (4 nM) of human TFPIα to normalize fibrin deposition is likely due to all endogenous murine TFPI isoforms (TFPIα, TFPIβ, and TFPIγ) being inhibited. To ensure that human TFPIα was capable of inhibiting murine FVIIa and that the anticoagulant effect seen in the mice was not simply attributable to FXa inhibition, we performed control CAT assays using murine plasma into which we added human TFPIα in the presence of an anti-human TFPI K1 monoclonal antibody that does not influence the FXa inhibitory function of TFPIα (see fig. S1). This anti-K1 antibody inhibited the anticoagulant function of human TFPIα in murine plasma, demonstrating that the anticoagulant function of human TFPIα in murine plasma is attributable to the inhibition of both murine FXa and murine FVIIa.

### Characterization of human C4BP-β chain as a specific inhibitor of murine protein S cofactor function

**Fig. 5.**
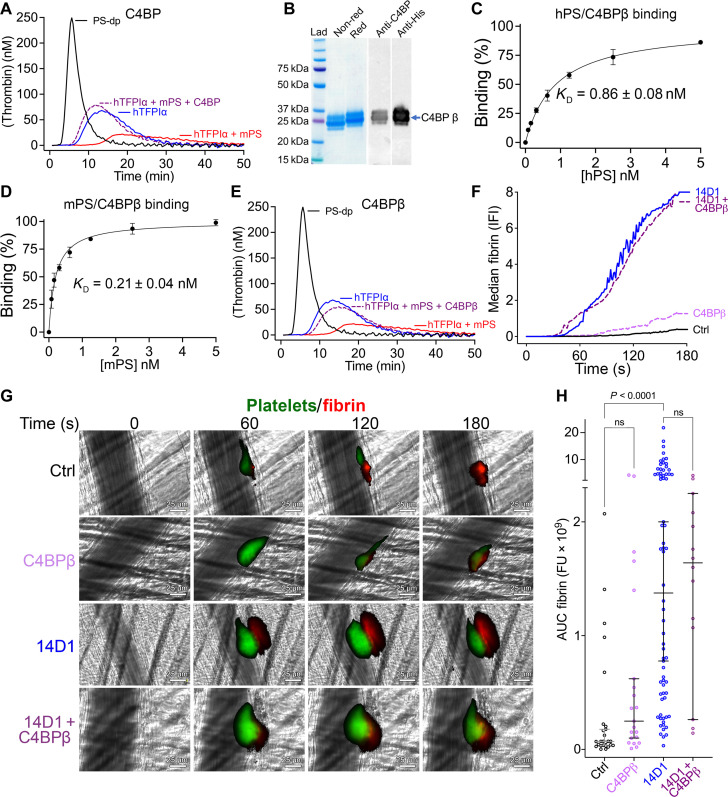
Recombinant C4BPβ binds and inhibits TFPIα cofactor function of mPS. (**A**) Inhibition of the hTFPIα cofactor function of mPS by plasma-purified human C4BP from was assessed using CAT assays in protein S–depleted plasma (PS-dp, which is codepleted in hTFPIα). Thrombin generation was initiated using 1 pM TF (Ctrl, black) containing 1 nM hTFPIα (blue), 1 nM hTFPIα, and 50 nM mPS (red) or 1 nM hTFPIα, 50 nM mPS, and 200 nM C4BP (purple dashed) (representative of *n* = 3). (**B**) Purified His-tagged CCP1-CCP2 domains of C4BP-β chain (termed C4BPβ) analyzed by SDS–polyacrylamide gel electrophoresis under reducing and nonreducing conditions and by Western blotting using anti-His and anti-C4BPβ antibodies. (**C** and **D**) Binding of human protein S [hPS (C)] or mPS (D) to immobilized C4BPβ are presented as normalized % maximal binding (mean of triplicates of *n* = 3). The binding affinities (*K*_D_) are presented as means ± SEM. (**E**) Inhibition of the hTFPIα cofactor function of mPS by C4BPβ was assessed as in (A) (graph representative of *n* = 3). (**F** to **H**) Laser-induced thrombosis in murine cremaster muscle arterioles was performed as in Fig. 4F. Representative images of mean fibrin deposition at 0 to 180 s in mice injected with control rat IgG (Ctrl), recombinant C4BPβ (300 nM), inhibitory rat anti-mTFPI (14D1), or 14D1 and C4BPβ. (G) Median fibrin deposition (IFI) over time in mice injected with control rat IgG (Ctrl, black; *n* = 26), C4BPβ (mauve; *n* = 21), inhibitory rat anti-mTFPIα (14D1, blue; 70), or 14D1 and C4BPβ (purple; *n* = 14). (H) For all thrombi, the total fibrin deposition (represented by the area under the curve, AUC fibrin) is plotted. Individual data are plotted with median ± 95% confidence interval. Data were compared by ANOVA using Dunn’s multiple comparisons test; *P* values <0.05 were considered significant.

We next aimed to specifically analyze the contribution of protein S cofactor function to TFPIα anticoagulant activity in vivo. For this, we exploited the natural high-affinity binding partner of protein S in humans, C4BP. To ascertain whether human C4BP binds to/blocks the TFPI cofactor function of murine protein S, as previously shown for human protein S (*15*), we performed CAT assays in protein S–depleted plasma supplemented with human TFPIα, murine protein S in the absence and presence of plasma-derived β chain–containing C4BP. In these assays, plasma-purified C4BP effectively blocked murine protein S–dependent enhancement of TFPIα anticoagulant function (Fig. 5A) in a similar manner to the antibody-mediated inhibition of human protein S (Fig. 1B) (*15*).

It is only the C4BP-β chain of C4BP that binds protein S. We, therefore, recombinantly expressed His-tagged complement control protein 1 (CCP1) and CCP2 domains of human C4BP-β chain in insect cells (herein termed C4BPβ). This material was purified (present in variably glycosylated forms) (Fig. 5B). To ascertain the affinity of recombinant C4BPβ for human and murine protein S, we performed enzyme-linked immunosorbent assay (ELISA)–based binding assays (Fig. 5, C and D) that revealed subnanomolar binding affinity to both human protein S (*K*_D_ = 0.86 ± 0.08 nM) and murine protein S (*K*_D_ = 0.21 ± 0.04 nM). Recombinant C4BPβ also effectively inhibited murine protein S cofactor function of human TFPIα in CAT assays (Fig. 5E), similar to plasma-derived β chain–containing C4BP (Fig. 5A) and antibody-mediated inhibition of human protein S (Fig. 1B).

Protein S has also been reported to allosterically regulate FIXa activity (*34*), although the molecular basis of this regulation currently remains unclear. To ascertain whether C4BPβ might influence hemostasis by impairing the ability of protein S to regulate FIXa, we performed CAT assays in normal human plasma in the presence of an inhibitory polyclonal anti-TFPI antibody in both the presence and absence of C4BPβ. Under these conditions, we detected no difference in thrombin generation, suggesting that C4BPβ does not impair this function of protein S in a manner that detectably alters thrombin generation in CAT assays (see fig. S2).

In addition to augmenting TFPIα function, protein S is a cofactor for activated protein C. Previous studies have revealed that human β chain–containing C4BP diminishes FVa inactivation by protein S/activated protein C by six- to eightfold (*35*). This is largely due to reduced proteolysis at R506 in FVa (*35*). Although this effect may be, at least in part, attributable to the steric hindrance associated with the large octomeric nature of C4BP, whether the C4BPβ alone influences activated protein C cofactor function of protein S was unclear. We therefore performed CAT assays in human plasma into which we titrated recombinant soluble thrombomodulin (0 to 20 nM). In the absence of C4BPβ, we saw a dose-dependent reduction in thrombin generation as soluble thrombomodulin concentration increased (see fig. S3A). This reduction was diminished in the presence of 300 nM C4BPβ (see fig. S3B), demonstrating that C4BPβ does partially impair protein S cofactor function for activated protein C to a similar extent as human β chain–containing C4BP (*35*).

To explore the influence of C4BPβ in the murine laser injury model, we injected 300 nM C4BPβ (final concentration) either alone or in combination with the inhibitory anti-murine K2 antibody (14D1). C4BPβ had no effect upon fibrin deposition in the presence of control IgG nor when given in combination with 14D1 (Fig. 5, F to H). These data reveal that C4BPβ does not, by itself, influence the hemostatic response in mice in the laser injury model. These findings are entirely consistent with our previous data demonstrating that inhibition of endogenous thrombomodulin in mice does not influence fibrin deposition in the acute response to the laser-induced injury (*28*) and that any effect of C4BPβ upon the endogenous protein C anticoagulant pathway does not majorly modify of the acute thrombotic response in this model. Thrombosis models that analyze thrombus formation and fibrin deposition over longer time frames are, of course, likely to be appreciably more sensitive to the actions of the endogenous protein C pathway.

### Role of protein S cofactor function in augmenting TFPIα anticoagulant function

**Fig. 6.**
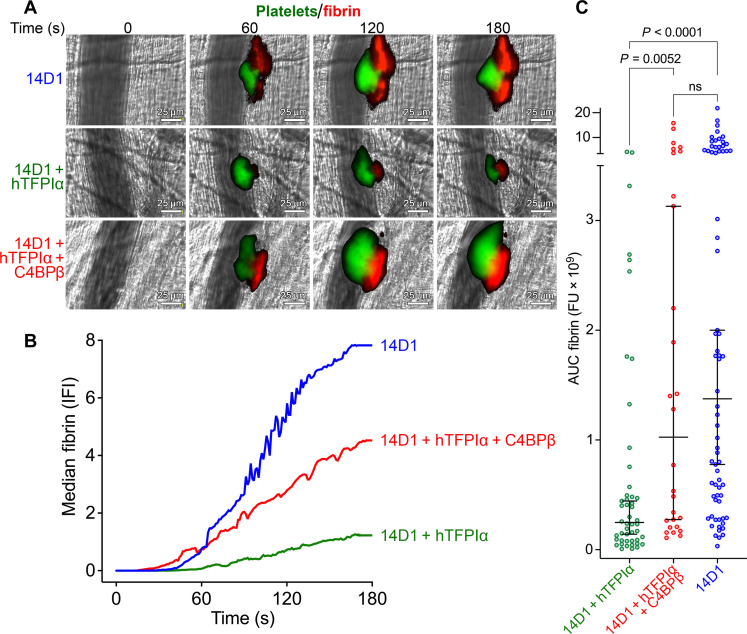
Importance of protein S as a cofactor for TFPIα anticoagulant function in vivo. Laser-induced thrombosis in murine cremaster muscle arterioles was performed using a mild laser injury following injection of anti–GPIbβ–DyLight 488 antibody to label platelets (green) and Alexa 647–labeled human fibrinogen (red) to measure fibrin deposition. Thrombus formation was monitored in real-time by intravital microscopy; see also accompanying movie S2. (**A**) Representative images of mean fibrin deposition at 0 to 180 s in mice injected with inhibitory rat anti-mTFPI (14D1); 14D1 and 4 nM hTFPIα; or 14D1, 4 nM hTFPIα, and 300 nM C4BPβ. (**B**) Median fibrin deposition (IFI) over time in mice injected with inhibitory rat anti-mTFPIα (14D1, blue; *n* = 70); 14D1 and 4 nM hTFPIα (green; *n* = 48); or 14D1, 4 nM hTFPIα, and 300 nM C4BPβ (red; *n* = 28). (**C**) For all thrombi, the total fibrin deposition (represented by the area under the curve,– AUC fibrin) is plotted. Individual data are plotted with median ± 95% confidence interval. Data were compared by ANOVA using Dunn’s multiple comparisons test; *P* values <0.05 were considered significant.

We then assessed whether blocking murine protein S with recombinant C4BPβ influenced the anticoagulant function of human TFPIα in mice. As before, all endogenous murine TFPI isoforms were blocked with 14D1 and normalized through the provision of 4 nM human TFPIα (Fig. 6, A to C). When mice were administered with 14D1, 4 nM human TFPIα, and 300 nM C4BPβ, there was an appreciable increase in fibrin deposition over time when compared to 14D1 and 4 nM human TFPIα (Fig. 6B and movie S2). When measuring the total fibrin deposition using the area under the fibrin deposition curves, mice injected with 14D1, 4 nM human TFPIα, and C4BPβ had significantly more fibrin deposition than those receiving 14D1 and 4 nM human TFPIα (*P* = 0.005). There was no significant difference between mice injected with 14D1, 4 nM human TFPIα, and C4BPβ and those injected with 14D1 alone (*P* = 0.71) (Fig. 6C), revealing that TFPIα function in vivo is highly dependent on protein S cofactor function (for platelet accumulation data for these experiments, please see fig. S4).

## DISCUSSION

In this study, we aimed to elucidate the contribution of protein S cofactor function to the anticoagulant actions of TFPIα in vivo. Since the original description of protein S as a cofactor for TFPIα by Hackeng and colleagues (*10*), there have been numerous reports of assays that are sensitive to, or help delineate the molecular basis of, protein S–dependent enhancement of TFPIα anticoagulant function (*7*, *8*, *10*, *11*, *15*, *17*, *29*, *32*). Of these, perhaps the most compelling are the plasma-based thrombin generation, or CAT, assays that reveal that protein S augments TFPIα function—seen primarily by extension of the lag time and reduction in the peak height (Fig. 1, A and B). These assays are particularly powerful as they are sensitive to, or can be made sensitive to, the function and/or amount of almost every coagulation factor/inhibitor. These assays also nicely distinguish between the different phases or coagulation (i.e., initiation and propagation). Despite this, translating CAT assay data to the physiological setting can be challenging (except perhaps in the more extreme deficiency states). For example, the contribution of TFPIα in CAT assays is markedly different when comparing assays using 1 pM versus 4 pM TF (*10*). How these concentrations compare to physiological TF exposure remains unclear. Blood flow is another important variable that is missing from CAT assays. In vivo, blood flow ensures constant provision of soluble coagulation factors/inhibitors and removes some of the activated factors from the site of clot formation, which alters coagulation kinetics. Platelets are another major variable often missing in CAT assays, generally replaced by coagulation-optimized phospholipid vesicles in suspension.

To more closely mimic physiological hemostasis, we developed a microfluidic hemostasis model using TF as the coagulation initiator and platelets rather than phospholipid vesicles. The major difference in our system is the use of VWF (as opposed to collagen) to recruit fresh platelets from whole blood immediately after blood sampling (Fig. 1C). Platelet binding to VWF facilitates formation of a uniform single layer of platelets that are not activated (*36*). Platelet binding to VWF alone does not induce activation, degranulation, or changes in phospholipid presentation. This is in contrast to the robustly activated microaggregates that form on collagen surfaces and that can occlude and alter channel flow patterns markedly. This means that platelet activation is primarily driven by thrombin following activation of coagulation by TF. For this, recalcified citrated plasma was perfused at low shear through the channel and TF-dependent fibrin deposition quantified. In these assays, blocking TFPI in plasma profoundly shortened the lag time (Fig. 1, C and D). A very similar effect was seen when protein S was blocked. This could suggest that under flow, there may be a greater dependency of TFPIα function on protein S to assist in its recruitment to platelet surfaces. Although this microfluidic assay has some advantages over CAT assays (as well as disadvantages), it still remains difficult to extrapolate these data to provide insight into the importance of protein S to TFPIα in vivo.

To address this, we developed tools to probe the TFPIα-protein S axis in mice. Because of the markedly reduced concentration of TFPIα in murine plasma (60 pM) and the elevated levels of TFPIγ (30 nM), which is not enhanced by protein S (*24*), we developed a model in which all murine TFPI isoforms were blocked to enable the anticoagulant function of human TFPIα to be more specifically assayed. We generated the 14D1 monoclonal antibody that specifically blocks murine TFPI K2 domain inhibitory function but does not cross-react with human TFPI (Figs. 2 and 3). Blocking endogenous murine TFPI caused a profound increase in fibrin deposition (and also platelet accumulation) in the laser injury model (Fig. 4), highlighting the importance of murine TFPI in regulating thrombus formation, similar to a previous report (*28*). In this model, we administered ~500 nM 14D1 to ensure that any TFPI that was released/exposed at sites of vessel damage was also efficiently and rapidly blocked. Our previous study revealed that reducing thrombin generation/fibrin deposition in this model increases platelet embolization, likely due to a combination of reduced thrombin-dependent platelet activation and platelet plug stabilization by fibrin. Consistent with this (although we did not formally quantify embolization), platelet accumulation in the presence of 14D1 increased significantly, but the effect of 14D1 on thrombus size/platelet accumulation was less marked than its effect upon fibrin deposition. Provision of human TFPIα dose-dependently reduced fibrin deposition and reduced platelet plug size, revealing that plasma TFPIα can compensate for the actions of TFPIβ and TFPIγ (Fig. 4). The supraphysiological concentration of 4 nM human TFPIα required to normalize fibrin deposition to the levels of control mice is likely due to the following: (i) 14D1 blocks all TFPI isoforms in mice (TFPIα, TFPIβ, and TFPIγ), which would in turn require increased human TFPIα concentrations to also compensate for the loss of their anticoagulant contributions and (ii) the *F5* gene in mice does not appear to have the splice sites that are responsible for the production of FV-short in humans, making it unlikely that mice express FV-short (although this has not been formally demonstrated). In humans, FV-short and protein S synergistically enhance human TFPIα. Moreover, FV-short also appears to maintain TFPIα in circulation. Because of the way in which our in vivo model of human TFPIα function has been established and the likely lack of endogenous FV-short in mice, this model could be ideally suited to now assess the ability of exogenous human FV-short to both influence the half-life and function of human TFPIα in vivo as well as its application to other models of thrombosis.

To specifically block protein S enhancement of TFPIα in mice, we attempted several different strategies. Although we generated a mouse anti-human TFPI K3 monoclonal antibody that efficiently inhibited protein S enhancement of FXa inhibition by TFPIα in vitro, it also appeared to block TFPIα inhibition of TF-FVIIa in the absence of protein S in CAT assays. We also developed rat monoclonal antibodies against the C-terminal laminin G_1_ and G_2_ domains of murine protein S, which harbors the TFPI K3 binding site, but despite extensive screening, we failed to identify antibodies that specifically blocked protein S–dependent enhancement of TFPIα. We, therefore, exploited the high-affinity binding of C4BPβ to human protein S, which has previously been demonstrated to block its ability to enhance human TFPIα function (*15*). As previously shown for human protein S (*15*), recombinant C4BPβ bound to murine protein S with subnanomolar affinity (Fig. 5, B to D) and effectively blocked the murine protein S cofactor function for human TFPIα in CAT assays (Fig. 5E).

Protein S can potentially modify the hemostatic response in distinct ways. It was recently reported that protein S can control FIXa function (*34*). Whether the binding of C4BPβ to protein S influences this function is unclear as how protein S controls FIXa activity mechanistically remains largely uncharacterized. At this time, there are no data to implicate the involvement of the C-terminal laminin G_1_ and G_2_ domains of protein S in inhibiting FIXa. However, we detected no influence of adding protein S alone to protein S–deficient plasma in the thrombin generation profile using 1 pM TF (Fig. 1A). Moreover, when we added C4BPβ to normal plasma containing anti-TFPI inhibitory antibodies, we detected no inhibitory effect upon thrombin generation, suggesting that C4BPβ unlikely influences this function of protein S. Consistent with this, when we injected C4BPβ alone to mice, we detected no effect on fibrin deposition in the presence or absence of endogenous TFPI function, strongly suggesting that C4BPβ unlikely has the ability to influence the hemostatic response in vivo in a protein S/FIXa–dependent manner.

We found that, in CAT assays containing soluble thrombomodulin, C4BPβ diminishes the activated protein C cofactor function of protein S, in a manner similar to plasma-derived β chain–containing C4BP, reported to be between six- to eightfold. This had the potential to confound the use of C4BPβ to specifically block the TFPIα cofactor function of protein S in vivo. However, we have previously demonstrated that, by blocking murine thrombomodulin with an inhibitory antibody, the local activation of endogenous protein C does not influence fibrin deposition in the acute laser injury model of thrombosis in mice. Consistent with this, recombinant C4BPβ did not, by itself, alter fibrin deposition in the laser-induced thrombosis model (Fig. 5, F to H). For these experiments, we injected a final concentration of 300 nM recombinant C4BPβ. The concentration of protein S in murine plasma is ~150 nM. Given the high-affinity interaction between C4BPβ and murine protein S, at 300 nM C4BPβ, essentially all murine protein S should be saturated with C4BPβ. This important control serves to demonstrate two contentions. First, this corroborates the contention that, in this model, any effect of C4BPβ on the protein C anticoagulant pathway does not modify fibrin deposition and, second, that the endogenous murine TFPIα-protein S pathway is not of major importance (Fig. 5, G and H). This latter contention is entirely consistent with the normal response/fibrin deposition in the laser injury using TFPIα-deficient mice (*27*). Mice have much lower plasma concentrations of TFPIα (60 pM) than humans (0.2 to 0.5 nM). This likely reduces the potency of this pool in hemostatic regulation in mice. TFPIα is present in murine platelets, which can be released following activation. These data therefore reveal that platelet TFPIα does not modify fibrin deposition in the laser injury model in mouse arterioles. Platelet TFPIα has been previously reported to influence platelet accumulation in electrolytic injury models of the femoral vein and carotid artery, but this effect was primarily observed after longer time frames than those measured in the laser injury model (*27*). Together, these control experiments reveal that C4BPβ is an appropriate strategy to block murine protein S cofactor function for TFPIα in a model that is not sensitive to any effects of C4BPβ upon the activated protein C– or FIXa-dependent actions of protein S.

Inhibition of murine protein S cofactor function with C4BPβ in the presence of human TFPIα significantly increased fibrin deposition, suggesting a major role for protein S in potentiating TFPIα function in vivo. Analysis of the total fibrin deposition (Fig. 6H) suggested that blocking protein S in the presence of human TFPIα increased fibrin deposition to similar levels seen in mice in the absence of human TFPIα. This finding is consistent with our microfluidic model data that also support the contention that the anticoagulant function of TFPIα under flow on platelet surfaces is largely dependent on protein S cofactor function. Whether this is attributable to differences in affinity of TFPIα and/or protein S for platelet versus phospholipid vesicle surfaces or associated with the ability of protein S to diminish the influence of flow on TFPIα inhibitory function remains to be determined. Despite this, it appears highly likely that TFPIα is only able to significantly contribute to hemostatic control at such low physiological concentrations in humans due to the effects of protein S cofactor function. These data also provide the potential to consider targeting the TFPIα cofactor function of protein S therapeutically. In patients with hemophilia, recent rebalancing strategies, particularly for those individuals that develop inhibitors, that aim to diminish endogenous anticoagulant pathways have been successfully trialed. Targeting TFPI using inhibitory antibodies is effective in reducing bleeding (*37**–**39*), but this has also resulted in thrombosis in a small number of cases (*40*). The reason for this may be that, as TFPI is the only endogenous inhibitor of TF-FVIIa, inhibition of all TFPI isoforms (TFPIα and TFPIβ), although effective, may enable the excessive persistence of the initiation of coagulation (as well as persistence of TF-FVIIa signaling). Our data demonstrate that targeting protein S cofactor function has a profound effect upon the efficiency of TFPIα function. This, however, would not completely prevent the inhibition of TF-FVIIa that may, in some cases, lead to thrombosis. Therefore, a potentially safer strategy might be to target the protein S enhancement of TFPIα, which is consistent with the hypothesis that more specific targeting of TFPIα function might be a safer approach (*41*).

## MATERIALS AND METHODS

### Recombinant proteins and antibodies

Murine C-terminally His-tagged protein S was expressed in human embryonic kidney (HEK) 293T cells and purified by a combination of barium citrate purification and anion exchange chromatography on a diethylaminoethyl (DEAE) column, as previously described for human protein S (*6*, *15*, *42*, *43*). Purified murine protein S was quantified using absorbance at 280 nm with a NanoDrop.

Human and murine TFPIα with a C-terminal His tag were expressed in HEK293T and purified on Ni^2+^-chelating HiTrap HP columns, followed by Heparin HiTrap HP column, as previously described (*15*). Full-length human TFPIα was quantified by ELISA, as previously described (*6*, *7*); the concentration of purified murine TFPIα was determined using absorbance at 280 nm with a NanoDrop and verified by functional comparison to human TFPIα in FXa inhibition assays. Before use, we verified that the C-terminal His tag does not influence TFPI anticoagulant function or its enhancement by protein S (fig. S5).

The isolated K1, K2, and K3 domains of murine TFPIα were expressed in *Drosophila* Schneider 2 insect cells and purified using a combination of Ni^2+^-chelating HiTrap HP and HiTrap Heparin HP columns. Full-length (protein S–free) C4BP was isolated from citrated human plasma, as previously described (*15*). The C-terminal His-tagged CCP1-CCP2 (Ser^1^-Ser^119^) domains of the C4BPβ was expressed in *Drosophila* Schneider 2 insect cells and purified on Cu^2+^- and Ni^2+^-charged HiTrap chelating HP columns followed by Superdex 200 Increase 10/300 GL column and quantified using absorbance at 280 nm with a NanoDrop, as previously described (*15*).

To generate rat monoclonal anti-murine K2 domain antibodies, rats were immunized three times with purified murine K2 (Fusion Antibodies). Following hybridoma generation and three rounds of affinity and functional selection, the 14D1 monoclonal was expressed from hybridoma cells in Dulbecco’s modified Eagle’s medium (Gibco Life Technologies) supplemented with 10% ultralow IgG fetal bovine serum (Sigma-Aldrich). Conditioned media were collected and 14D1 purified using a HiTrap Protein G HP column, buffer-exchanged to 20 mM Hepes and 150 mM NaCl (pH 7.4). The concentration of 14D1 was estimated using absorbance at 280 nm with a NanoDrop. Rat anti-murine protein S monoclonal antibodies were generated and selected as above using recombinant murine protein S to immunize rats (Fusion Antibodies).

### Ex vivo flow assay

Vena8 microfluidic channels were coated with 50 nM VWF and 20 pM TF in phosphate-buffered saline overnight at 4°C. Channels were washed with modified Hepes-Tyrodes (HT) buffer and blocked with 1% bovine serum albumin (BSA) in HT buffer. Fresh citrated human whole blood collected in corn trypsin inhibitor (18 μg/ml; Enzyme Research Laboratories) was incubated at 30°C with 2.5 μM DiOC6 for 10 min and perfused through the microfluidic channels at 1000 s^−1^. Platelet monolayer adhesion was visualized using a Zeiss Vert A1 microscope with a 20× objective, and images (focused on a single section) were recorded every second for 5.5 min with a camera format 2 × 2 binning. Channels were washed by perfusing 0.1% BSA in HT buffer for 2 min. Recalcified pooled human plasma (5 mM CaCl_2_ and 3.8 mM MgCl_2_—final free concentrations) containing Alexa 594–labeled fibrinogen (60 μg/ml; Invitrogen) and either control IgG (mix of mouse and rabbit IgG) or a mix of anti-human TFPI K1, K2, and C-terminal tail monoclonal antibodies (Sanquin, 133 nM each) or 3.6 μM rabbit anti-human protein S (Agilent) was immediately perfused at a 100 s^−1^ shear rate for 10 min. Fibrin deposition was visualized as above. Sum fluorescence intensity was calculated using Slidebook 6.0 software (3i). The lag time was designated as the time to 2% maximal fibrin fluorescence in the anti-TFPI condition paired with each experimental condition. The velocity of fibrin deposition was derived using the rate of change in fibrin fluorescence during the linear phase of fibrin deposition. Data were compared using one-way analysis of variance (ANOVA) with Dunnett’s multiple comparison test in GraphPad software (Prism).

### FXa inhibition assays

The activity of 0.5 nM FXa (Enzyme Research Laboratories) was measured using 200 μM chromogenic substrate S-2765 (Chromogenix), 4 μM phospholipid vesicles [20:20:60 molar ratio of 1,2-dioleoyl-*sn*-glycero-3-phosphoethanolamine, 1,2-dioleoyl-*sn*-glycero-3-phosphoserine, and 1,2-dioleoyl-*sn*-glycero-3-phosphocholine; Avanti Polar Lipids, prepared by extrusion as previously described (*43*)] in 20 mM tris and 150 mM NaCl [tris-buffered saline (TBS)] containing 5 mM CaCl_2_, which was followed in real time by measuring the absorbance at 405 nm for 60 min. FXa inhibition by human and murine TFPIα in the presence and absence of human plasma purified protein S (C4BP-free) (Enzyme Research Laboratories) and murine protein S (160 nM) were monitored as described previously (*7*). We assessed the ability of 14D1 (80 nM) to block either human TFPIα or murine TFPIα (8 nM) inhibition of FXa in the same way. 14D1 (80 nM) was also tested in reactions containing either 2 nM human TFPIα or murine TFPIα in the presence of 50 nM murine protein S. To measure the potency of 14D1 in inhibiting murine TFPIα (IC_50_), we performed experiments in the presence of 5 nM murine TFPIα containing increasing concentrations of 14D1 (0 to 80 nM).

### Thrombin generation assays

Thrombin generation was monitored in citrated normal human plasma or protein S–depleted human plasma (Enzyme Research Laboratories) using CAT, as described previously (*6*, *7*, *43*). In all experiments, 1 pM TF, 4 μM phospholipid vesicles, and 5 mM CaCl_2_ were used. To assess the effect of endogenous TFPIα and protein S upon thrombin generation in normal human plasma, polyclonal sheep antibodies against human TFPI (80 μg/ml; Prolytix) and polyclonal anti-human protein S (420 μg/ml; Agilent), respectively, were preincubated with the plasma for 10 min before the initiation of coagulation. Formal titration of the polyclonal anti-TFPI antibody in CAT assays was performed to ensure complete TFPI inhibition. The inhibitory effect of the protein S antibodies at 420 μg/ml was previously reported (*6*, *7*, *10*, *44*). The effect of human TFPIα and protein S upon thrombin generation was also studied in protein S–depleted plasma, also codepleted of full-length TFPIα (*15*), by preincubating plasma with 1 nM recombinant human TFPIα and 50 nM human or murine protein S for 10 min before the initiation of coagulation. To specifically block the enhancement of human TFPIα by murine protein S, full-length plasma-purified, protein S–free C4BP (200 nM) (*42*) or recombinant His-tagged C4BPβ (200 nM) were mixed with human TFPIα and murine protein S in protein S–depleted plasma for 10 min before the initiation of coagulation.

### Plate-binding assays

The affinity (*K*_D_) between human or murine protein S and the His-tagged C4BPβ was measured using ELISA-based plate-binding assays. For this, purified C4BPβ (2 μg/ml) in 50 mM sodium bicarbonate buffer (pH 9.6) were adsorbed overnight at 4°C onto MaxiSorp wells (Thermo Fisher Scientific). Wells were washed and blocked with TBS/5 mM CaCl_2_/5% gelatin (pH 7.4, blocking buffer). The wells were washed, and 100 μl of murine protein S or human protein S (0 to 5 nM) in blocking buffer was added and incubated for 1.5 hours. Uncoated, blocked wells were used to control for nonspecific binding. After washing, two-step detection of bound murine protein S was performed using an in-house rat anti-murine protein S monoclonal antibody (2 μg/ml) followed by horseradish peroxidase (HRP)–conjugated goat anti-rat IgG (2 μg/ml; Sigma-Aldrich). Detection of bound human protein S was performed using polyclonal rabbit anti-human protein S antibodies (2 μg/ml) followed by HRP-conjugated polyclonal goat anti-rabbit IgG (2.5 μg/ml; Agilent). After subtracting the signal from control wells, the data were fitted in the GraphPad software (Prism) using the one-site binding equation: *B* = *B*_max_ × [PS]/(*K*_D(App)_ + [PS]), where *B* is the percentage-specific binding, *B*_max_ is the maximal binding, and *K*_D(App)_ is the apparent equilibrium dissociation constant. Data are presented as means ± SD of three separate experiments. The affinity (*K*_D_) between 14D1 and the murine TFPIα was measured as above using purified murine TFPIα (1 μg/ml) adsorbed to wells and 100 μl of purified 14D1 (0 to 5 nM) in blocking buffer. Detection of bound 14D1 was performed using HRP-conjugated goat anti-rat IgG (Sigma-Aldrich), and data were fitted as above. The affinity (*K*_D_) between 14D1 and the isolated murine K2 domain was measured using essentially the same protocol but with His-tagged murine K2 (0.1 μg/ml) in TBS (pH 7.4) immobilized on preblocked Ni^2+^–nitrilotriacetic acid plates, and 1% BSA was used as blocking agent in all buffers instead of gelatin.

### Laser-induced thrombosis model

All procedures were performed with U.K. Home Office approval in accordance with the Animals (Scientific Procedures) Act of 1986. C57Bl6/J male mice (7 to 10 weeks) were anesthetized by intraperitoneal injection of ketamine (75 mg/kg) and medetomidine (1 mg/kg). Anesthesia was maintained with pentobarbital (5 mg/kg), as required. The cremaster muscle was exteriorized and the connective tissue was removed, after which the cremaster muscle was affixed over a glass slide; the muscle preparation was hydrated throughout with saline. Control rat IgG (2 μg/g), inhibitory rat anti-murine TFPI (14D1; 2 μg/g), recombinant human TFPIα (1 to 4 nM final concentration), or recombinant C4BPβ (300 nM final concentration) and DyLight 488 anti-GPIbβ antibody (0.15 μg/g; for platelet labeling; Emfret Analytics) and Alexa 647 fibrinogen [5% (w/v) blood volume; Invitrogen) were infused into the mouse circulation through a jugular vein cannula before the injury (performed using a laser ablation system, Ablate!; 3i). Injuries were scored on a four-point scale. More severe injuries that punctured the vessel were excluded. Mouse blood volumes were estimated using the calculation of 80 μl/g to determine final concentrations of human TFPIα and C4BPβ, and the infusion volume was normalized according to body weight—For these experiments, the operator was blinded to the contents of the injection mixture until after analyses and quantitation had been performed. Fibrin deposition following thrombus formation was visualized and captured using VIVO intravital imaging system (Intelligent Imaging Innovations 3i). Images were analyzed using Slide book 6 software (Intelligent Imaging Innovations). Data were compared with GraphPad software (Prism) using ANOVA with Dunn’s multiple comparisons test.
