# Supplementary material for: TFPIα anticoagulant function is highly dependent on protein S in vivo
Source: Sci Adv. 2024 Feb 2;10(5):eadk5836. doi: 10.1126/sciadv.adk5836 (PMC11809661; doi:10.1126/sciadv.adk5836)
Supplement: Supplementary file 1 — Supplementary Methods Figs. S1 to S5 Legends for movies S1 and S2 [file sciadv.adk5836_sm.pdf]

Science Advances
AAAS

Supplementary Materials for

TFPIα anticoagulant function is highly dependent on protein S in vivo

Anastasis Petri *et al.*

Corresponding author: James T. B. Crawley, j.crawley@imperial.ac.uk

Sci. Adv. **10**, eadk5836 (2024)
DOI: 10.1126/sciadv.adk5836

The PDF file includes:

Supplementary Methods
Figs. S1 to S5
Legends for movies S1 and S2

Other Supplementary Material for this manuscript includes the following:

Movies S1 and S2

Supplementary Methods

Murine plasma CAT assays

Thrombin generation was monitored in citrated murine plasma containing in 65 μg/ml corn trypsin inhibitor (to inhibit contact activation) using CAT. In all experiments, 1 pM TF, 50 μM phospholipid vesicles and 5 mM $CaCl_2$ were used in dilute murine plasma (20 μl diluted in TBS containing 0.5 % BSA). Endogenous murine TFPI was inhibited by preincubation of 277 nM inhibitory anti-murine K2 antibody (14D1) with the plasma for 10 minutes prior to the initiation of coagulation. The effect of human TFPIα on thrombin generation was studied by pre-incubating plasma with 5 nM recombinant human TFPIα for 10 minutes prior to the initiation of coagulation in the absence and presence of 111 nM anti-human K1 antibody (Sanquin). Thereafter, thrombin generation was monitored as previously described for human plasma CAT assays. (*6, 7, 43*)

Supplementary Figures

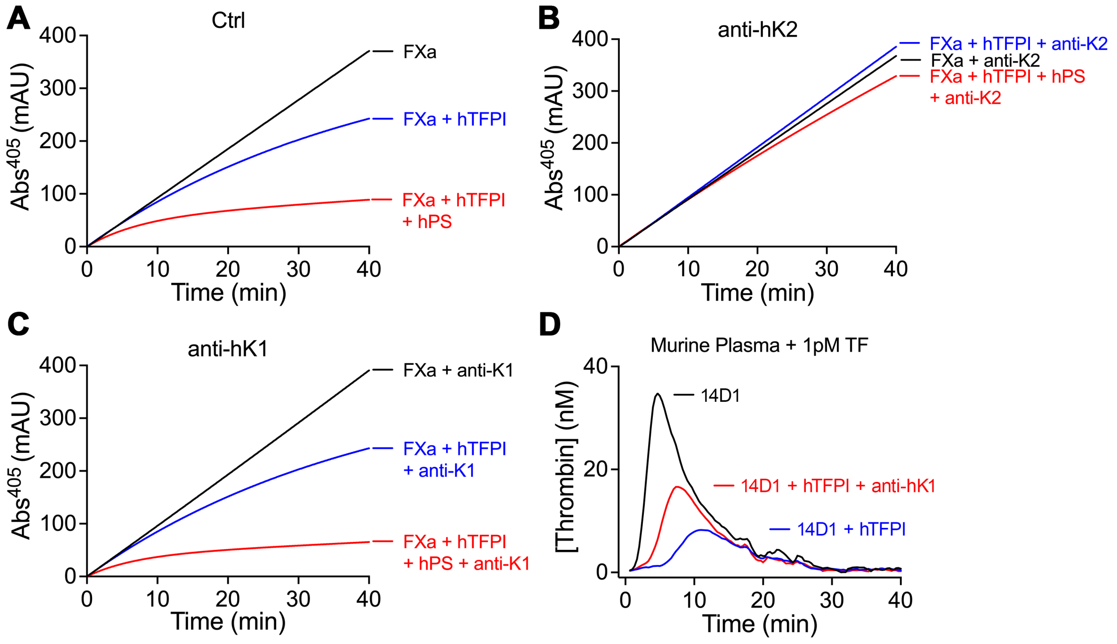

Fig. S1. Human TFPIα anticoagulant function in murine plasma includes inhibition of murine FVIIa.

A) To determine whether human TFPIα inhibits murine FVIIa, we first performed FXa activity assays in the presence of human TFPIα (blue) ± human protein S (red). **B**) FXa activity assays as in (A) except also in the presence of an inhibitory anti-human K2 domain monoclonal antibody (40 nM) that blocks FXa inhibition. **C**) FXa activity assays as in (A) except in the presence of an anti-human K1 domain monoclonal antibody. The anti-K1 antibody does not impair FXa inhibition by TFPIα in the absence or presence of protein S. **D**) To test whether inhibition of murine FVIIa by human TFPIα occurs, we performed CAT assays using 1 pM TF in diluted murine plasma in the presence of the inhibitory anti-murine K2 antibody (14D1) to inhibit endogenous murine TFPI (black). Addition of 5 nM human TFPIα (blue) reduced thrombin generation and extended the lag time. Addition of 5 nM TFPIα and the monoclonal anti-human K1 domain antibody reduced the inhibitory function of human TFPIα demonstrating that the anticoagulant function of human TFPIα includes inhibition of murine FVIIa bound to TF.

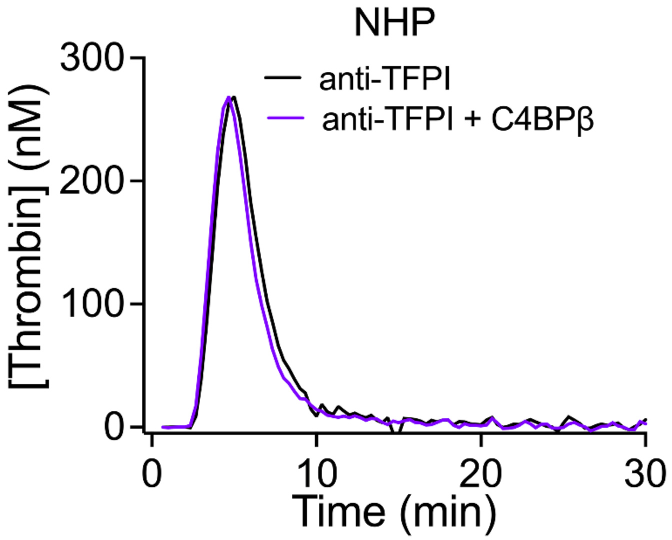

Fig. S2. C4BPβ does not influence thrombin generation in the absence of the protein C or TFPI anticoagulant pathways.

To test whether recombinant C4BPβ influences thrombin generation in a manner that is independent of the protein C or TFPI anticoagulant pathways, we performed CAT assays using 2 pM TF in normal human plasma (NHP). We inhibited TFPI by the addition of a polyclonal anti-TFPI antibody (black). Addition of 300 nM C4BPβ (purple) had no effect upon thrombin generation, suggesting that any effect of protein S that is independent of TFPI and protein C (e.g. FIXa inhibition) is not influenced by C4BPβ.

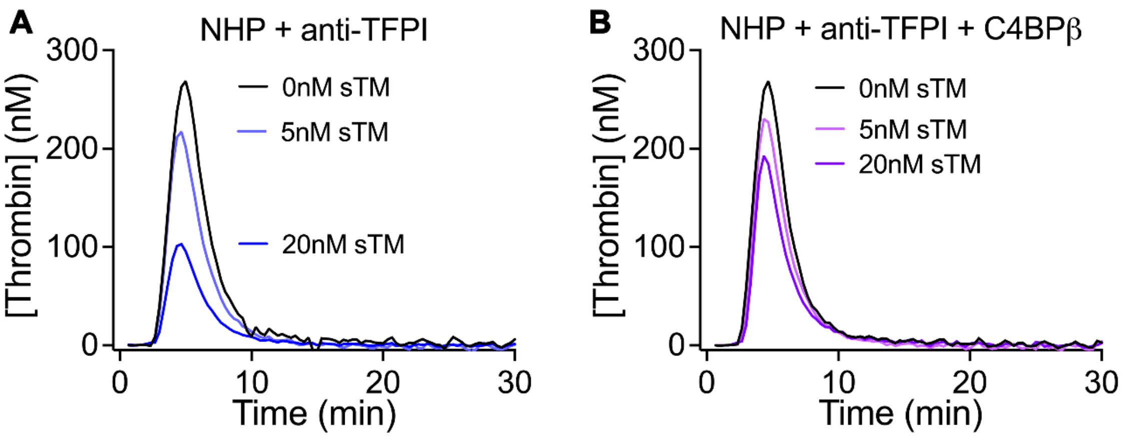

Fig. S3. Recombinant C4BPβ diminishes the activated protein C cofactor function of protein S.

A) To test whether C4BPβ influences the protein C anticoagulant pathway, we performed CAT assays in normal human plasma (NHP) containing an inhibitory polyclonal anti-TFPI antibody containing increasing concentrations (0-20 nM) of soluble thrombomodulin (sTM). As sTM concentration was increased, the endogenous protein C activation reduced thrombin generation. **B**) CAT assays as in (A) except also containing 300 nM C4BPβ. Recombinant C4BPβ reduced the anticoagulant function of the endogenous protein C pathway, suggesting that it partially inhibits the activated protein C cofactor function of protein S.

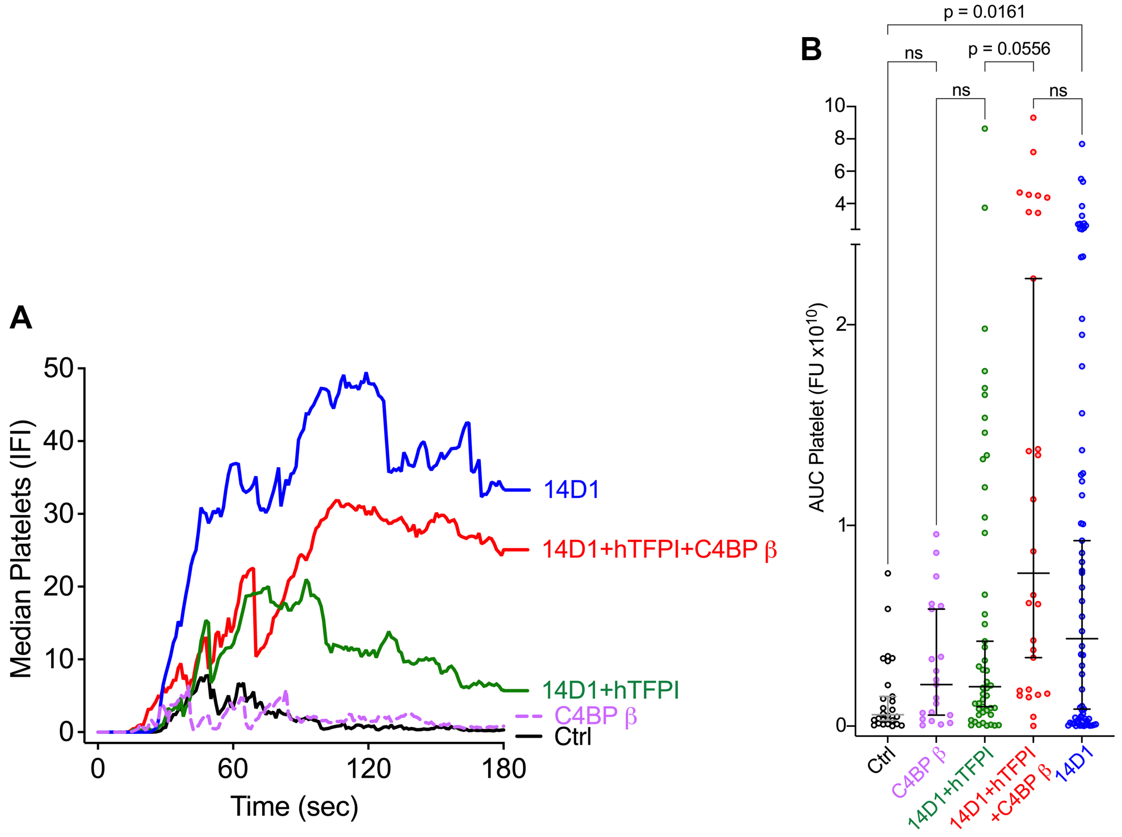

Fig. S4. Recombinant C4BPβ diminishes the activated protein C cofactor function of protein S.

A) Median platelet deposition (IFI) over time in mice injected with control rat IgG (Ctrl – black; n=26), inhibitory rat anti-mTFPI (14D1 - blue; n=70), 14D1 and 4 nM hTFPIα (green; n=48) or 14D1, 4 nM hTFPIα and 300 nM C4BPβ (red; n=28) and C4BPβ alone (purple; n=14). **B**) For all thrombi, the total platelet deposition (represented by the area under the curve – AUC Platelet) is plotted. Individual data are plotted with median ±95% confidence interval. Data were compared by ANOVA with a Dunn's multiple comparison test; p values <0.05 were considered significant.

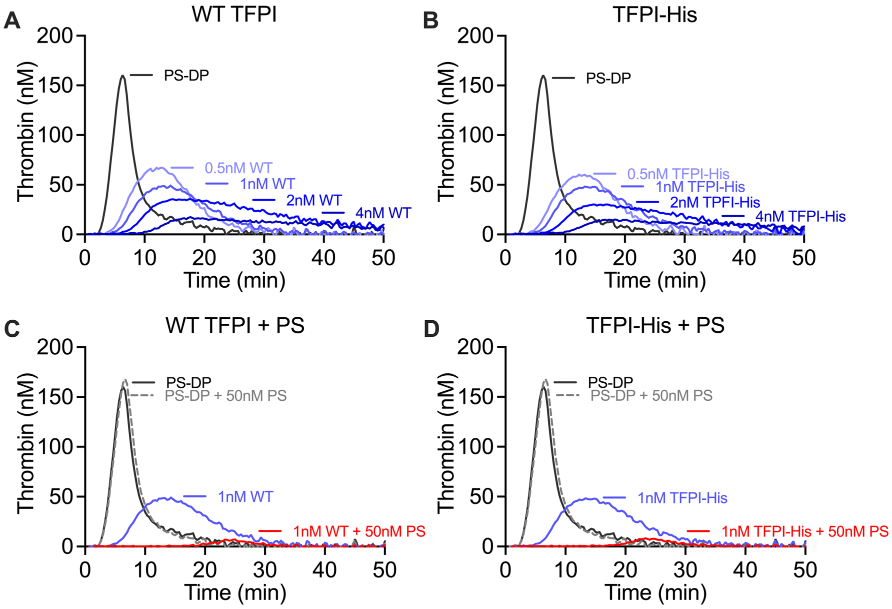

Fig S5. Human TFPIα anticoagulant function is unaltered by a C-terminal His tag.

A & B) Recombinant WT TFPIα or TFPIα with a C-terminal His tag (TFPI-His) were purified and quantified relative to each other by ELISA. Thereafter, their anticoagulant functions were assayed by titrating increasing concentrations (0.5-4 nM) of each into protein S-depleted plasma (PS-DP). Thrombin generation was initiated with 1 pM TF and measured using CAT assays. **C & D**) To assess whether the C-terminal His tag on TFPI-His influences protein S cofactor function, CAT assays were repeated as in A&B, except using on 1 nM WT TFPI/TFPI-His in the presence and absence of 50 nM protein S. In each of these assays, TFPI-His behaved indistinguishably from WT TFPI.

Legends for Supplementary Movies

Movie S1: Comparison of laser-induced thrombus formation in mice ±14D1 ±hTFPIα.
Laser-induced thrombosis in murine cremaster muscle arterioles was performed using a mild laser injury following injection of anti-GPIbβ-DyLight 488 antibody to label platelets (green) and Alexa 647-labelled human fibrinogen (red) to visualize fibrin deposition. Thrombus formation was monitored in real time by intravital microscopy. Representative movies of the fibrin/platelet deposition from 0-220 s in mice (C57Bl6/J) injected with control rat IgG, inhibitory rat anti-mTFPIα (14D1) or 14D1 + 4 nM recombinant hTFPIα.

Movie S2: Importance of protein S as a cofactor for TFPIα anticoagulant function *in vivo*.
Laser-induced thrombosis in murine cremaster muscle arterioles was performed using a mild laser injury following injection of anti-GPIbβ-DyLight 488 antibody to label platelets (green) and Alexa 647-labelled human fibrinogen (red) to visualize fibrin deposition. Thrombus formation was monitored in real time by intravital microscopy. Representative movies of the fibrin/platelet deposition from 0-220 s in mice (C57Bl6/J) injected with 14D1 + 300 nM C4BPβ, 14D1 + 4 nM hTFPIα or 14D1 + 4 nM hTFPIα + 300 nM C4BPβ.
